# MEASURING PERSONALITY IN DAILY LIFE: EVIDENCE FROM AN AGE-HETEROGENEOUS ADULT SAMPLE

**DOI:** 10.1093/geroni/igac059.2374

**Published:** 2022-12-20

**Authors:** Giselle Ferguson, Giancarlo Pasquini, Martin Sliwinski, Stacey Scott

**Affiliations:** Stony Brook University, Stony Brook, New York, United States; Stony Brook University, Port Jefferson Station, New York, United States; The Pennsylvania State University, University Park, Pennsylvania, United States; Stony Brook University, Stony Brook, New York, United States

## Abstract

Research on personality has theorized that repeated short-term experiences can lead to changes in personality traits across years or decades. Whereas much research on these short-term experiences relevant to personality has been done in samples of college students, this study intended to measure personality-relevant short-term experiences among an age-heterogeneous sample of adults. As part the Effects of Stress on Cognitive Aging, Physiology, and Emotions study, 260 participants (Mage=46.49 years, range=25-65 years) completed a measure of Big Five personality traits before completing a 14-day ecological momentary assessment (EMA) period during which participants reported their momentary negative and positive thoughts, emotions, and social interactions up to six times per day on study-provided smartphones. We hypothesized that these EMA reports could be used as daily markers of trait extraversion and trait neuroticism such that these daily experiences could be interpreted as manifestations of personality traits in daily life. Results of parallel multilevel confirmatory factor analyses showed good model fit (extraversion: CFI=0.96; TLI=0.95; RMSEA=0.03; neuroticism: CFI=0.95; TLI=0.94; RMSEA=0.04). For both extraversion and neuroticism, the latent trait factor and the latent daily-marker factor were positively correlated (extraversion: r=0.36; SE=0.07; p<.001; neuroticism: r=0.45; SE=0.07; p<.001). Results suggest that among an age-heterogeneous adult sample, momentary thoughts, feelings, and behaviors across a two-week period represented expressions of extraversion and neuroticism in daily life. Measuring these short-term experiences is meaningful for understanding how personality changes across adulthood, and future work can use longitudinal data to test if daily markers of personality are sensitive to fluctuations and changes in personality.

